# *Lobelia hongiana* (Campanulaceae), a new species from Guangxi, China

**DOI:** 10.3897/phytokeys.95.20245

**Published:** 2018-01-30

**Authors:** Zhi-Zhong Li, Neng Wei, Yan Liu, Jin-Ming Chen, Guang-Wan Hu, Qing-Feng Wang

**Affiliations:** 1 Wuhan Botanical Garden, Chinese Academy of Sciences, Wuhan, CN-430074, China; 2 Sino-Africa Joint Research Centre, Chinese Academy of Sciences, Wuhan, CN-430074, China; 3 University of Chinese Academy of Sciences, Beijing, CN-100049, China; 4 Guangxi Institute of Botany, Chinese Academy of Sciences, Guilin, CN-541006, China

**Keywords:** *Hypsela*, *Lobelia chinensis, Lobelia loochooensis*, Southern China

## Abstract

*Lobelia hongiana*, a new species of Campanulaceae from Guangxi, South China, is described and illustrated here. This new species is most similar to *L. chinensis* and *L. loochooensis*, but differs by its elliptic-obovate or oblanceolate leaf, 2.5–3 mm long greenish-carmine hypanthium, 5 or 6 calyx lobes, purplish-white corolla, with yellowish-green blotches at the base of lower lobes, glabrous filaments, 7–8 mm long broadly obconic capsule. Molecular phylogenetic analysis has been conducted based on ITS and two chloroplast sequences (*atpB* and *rbcL*) and 14 taxa in *Lobelia* are included. *L. hongiana* is well supported as a new species by the evidence from both morphology and molecular phylogeny.

## Introduction

*Lobelia* Linnaeus (1753: 929) (Campanulaceae) is mainly distributed in tropics and subtropics (Lammers 2011). Wimmer (1943, 1953, 1968) proposed the first comprehensive classification system of this genus, which was mainly based on some morphological characters. Subsequently, Murata (1995) and Lammers (2011) improved the classification system using more morphological characters. With over 400 species, *Lobelia* is the second largest genus in Campanulaceae (*Campanula* Linnaeus (1753: 163) is the largest one) and it was classified as 18 sections based on the morphological characters and molecular analyses (Lammers 2011; Chen et al. 2016). Of these, there are 23 species (with six endemic species), belonging to five sections, which have been recorded in China (Hong and Lammers 2011).

During a fieldwork in Huixian town of Guangxi Zhuang Autonomous Region in June 2016, some interesting specimens of *Lobelia* were collected near a local crops field. The leaf shape and flower characters of these individuals were distinctly different from those of the other described *Lobelia* in China. Besides the collected specimens of this unknown *Lobelia*, some individuals were also transplanted in the greenhouse of Wuhan Botanical Garden for further observations. Based on careful observation on morphological characters, literature consulting and specimen comparisons, it was found that these specimens should be a new species, belonging to L. sect. Hypsela (C. Presl) Lammers in Hong and Lammers (2011: 555). Morphologically, this new species is similar to *L. chinensis* Loureiro (1790: 514) as well as *L. loochooensis* Koidzumi (1929: 406) that is endemic to Okinawa, Japan. A molecular phylogeny using the combined ITS, *atpB* and *rbcL* dataset also supported these specimens as a separate species. In this study, therefore, the new species was named as *Lobelia hongiana* Q.F.Wang & G.W.Hu.

## Materials and methods

### Morphological observation

Morphological descriptions and comparisons are based on observations of *Lobelia* specimens from the herbaria of GXMG, HIB, IBSC, KUN, PE and literature. There are eight species of L. sect. Hypsela recorded in Southern and Eastern Asia (Lammers 2011), *L. archboldiana* (Merr. & L.M. Perry) Moeliono (1960: 131), *L. brachyantha* Merr. & L.M. Perry (1941: 385), *L. conferta* Merr. & L.M. Perry (1949: 59), *L. donanensis* P. Royen (1966: 305), *L. nummularia* Lam. (1792: 589), *L. victoriensis* P. Royen (1978: 118), *L. chinensis* and *L. loochooensis*. The taxonomic status of the eight species was examined by checking the type specimens from JSTOR Global Plants (http://plants.jstor.org/) and the protologue.

### Phylogenetic analysis

Two individuals were used from Huixian town, Guangxi Zhuang Autonomous Region, China to assess the phylogenetic position. A total of 14 closed taxa as ingroups and one *Trachelium* species as outgroup were used in phylogenetic analyses. Total genomic DNA was extracted from the fresh material according to Chen et al. (2016). Six sequences from two individuals of the new species were newly generated in this study and the other sequences were downloaded from NCBI (https://www.ncbi.nlm.nih.gov/) (Table 1). The primers were obtained following Haberle et al. (2009, *atpB* and *rbcL*) and Chen et al. (2016, ITS). PCR amplification, sequencing and sequence assembly were implemented following Chen et al. (2016). The best model of nucleotides substitution was selected using jModeltest 2.1.4 (Darriba et al. 2012) with the Akaike Information Criterion (AIC). The Maximum Likelihood (ML) analysis was obtained using RAxML version 8.1.24 (Stamatakis 2006), with separate partitions for the nuclear and plastid data using 1000 bootstrap replicates. The Bayesian Inference (BI) was performed by MrBayes version 3.2.6 (Ronquist and History 2015). Monte Carlo Markov chains were run for 10 million generations with sampling every 5,000 generations. The default setting was used for chain heating (temp = 0.2). The first 10 % of trees were discarded as burn-in and the remaining trees were combined to estimate posterior probability (PP) and other settings following Jin et al. (2016).

**Table 1.** GenBank accession numbers for sequence data of *Lobelia hongiana* used in this study. (“–” indicates not accessible).

Species	ITS	atpB	rbcL
*Rachelium caeruleum*	DQ304570	EU437661	EU713435
*Lobelia urens*	–	–	HM850130
*Lobelia linnaeoides*	–	EF694723	EF694723
*Lobelia macrodon*	AY568734	EF694736	EF694736
*Lobelia roughii*	–	EF694738.1	EF694738.1
*Lobelia oligophylla*	–	–	DQ356159
*Lobelia angulata*	AY362767	AJ235524.1	MF061180
*Lobelia nummularia*	–	MF061203.1	AB645964
*Lobelia purpurascens*	AY568729	–	DQ356160
*Lithotoma petraea*	–	KY354215	KY354215
*Lobelia chinensis*	KT957582	KC146452	KC146532
*Lobelia arnhemiaca*	–	EF694737	EF694737
*Isotoma fluviatilis*	AY644648	EF999977	DQ356161
*Lobelia loochooensis*	–	–	AB645961
*Lobelia hongiana*_1	MF580388	MF580392	MF580390
*Lobelia hongiana*_2	MF580389	MF580393	MF580391

## Results and discussion

The Bayesian tree showed that the new species is well supported as sister to *L. loochooensis* (ML bootstrap values = 96, PP = 1.00) which placed it in L. sect. Hypsela. Evidence from molecular phylogeny supports *L. hongiana* as an independent taxon, with *L. loochooensis* as the sister taxon. This study also made the supposition that L. sect. Hypsela originated from Australia and dispersed to New Zealand, Ryukyus and Southern China (Kokubugata et al. 2012, Chen et al. 2016).

*Lobelia hongiana* has the following characters, including its solitary flowers in the axils of leaves, a sub-bilabiate corolla with lobes longer than the tube, anthers with a single elongate bristle at the apex of each of the ventral pairs and seed coat reticulate. All of these characters group it into L. sect. Hypsela. In this section, this new species is most similar to *L. chinensis* and *L. loochooensis*, but the differences amongst them are also dominant (Table 2). Compared with *L. chinensis*, it has a smaller leaf, shorter hypanthium, calyx lobe 5 or 6, shorter than hypanthium, purplish-white corolla, corolla lobe not spreading in a plane on anterior side and shorter glabrous filaments. Compared with *L. loochooensis*, it has a prostrate stem, elliptic-obovate or oblanceolate leaf, longer pedicels, longer greenish-carmine hypanthium, its calyx lobes are shorter than hypanthium, longer corolla, bearing tufts of filiform hairs at 3 dorsal anther tubes and longer broad obconic capsule. Combined with morphological and phylogenetic analyses, *L. hongiana* is confirmed as new to science.

**Table 2.** Characters distinguishing *Lobelia hongiana*, *L. chinensis* and *L. loochooensis*. (The character information of *L. loochooensis* is mainly based on Murata (1992); “–” indicates the description of *L. loochooensis* is not yet accessible.)

Characters	*Lobelia hongiana*	*L. chinensis*	*L. loochooensis*
Stem	Decumbent	Decumbent	Prostrate
Leaf	Elliptic-obovate or oblanceolate, slightly thick, 2.2–9 × 1.6–5 mm, sessile or to 2mm, apex obtuse, margin usually sinuate-dentate or sub-entire	Narrowly elliptic, elliptic or lanceolate, thin, 7–26 × 1.5–7 mm, sessile or to 1 mm, apex acute or acuminate, margin entire or obviously serrate at upper part	Orbicular to broadly obovate to sub-obtriangular, thick, 5–7 mm in diam., almost sessile, apex rounded, margin entire or tridentate at upper part
Flower	Solitary, pedicel 1.3–4.4 cm long	Solitary at upper part of stem, pedicel 1.2–6.5 cm long	Solitary, pedicel 1–2.5 cm long
Hypanthium	2.5–3 mm long, greenish carmine	3–5 mm long, green	Ca. 1 mm long, yellowish green
Calyx lobes	5 or 6, lanceolate or sometimes unevenly bifid, 1.4–2 mm long, shorter than hypanthium, 1 or 2 pair(s) of denticles	5, lanceolate, 3–5 mm long, as long as hypanthium, 1 pair of denticles	5, narrowly triangular, 1.5 mm long, longer than hypanthium, –
Corolla	Purplish-white, 9–13 mm long, sub-bilabiate, lobes 5, gradually recurved when open, lobes equal or subequal, ovate-lanceolate	Rose, white or bluish, 10–15 mm long, unilabiate, lobes 5, all spreading in a plane on anterior side, lateral 2 lanceolate or oblanceolate, central 3 elliptic	White to pale violet, 8–9 mm long, sub-bilabiate, lobes 5, gradually recurved when open, lobes equal or subequal, oblong-lanceolate
Lower/Central lobes	Yellowish-green blotches at the base, apex recurved, covered sparsely white villous, without vein	Green blotches with yellow margin at the base, apex incurved, glabrous, with purple veins	Blue blotches at the base, apex recurved, glabrous, with blue veins
Filament	Ca. 4 mm long, glabrous	6–8 mm long, the two anterior ones hairy	Ca. 3 mm long, sparsely hairy
Anther	Tube 1.1–1.5 mm, bearing tufts of filiform hairs at 3 dorsal anther tubes	Tube 2–2.5 mm, 3 dorsal anther tubes sparsely villous or glabrous	Tube ca. 1 mm long, 3 dorsal anther tubes glabrous
Pistil	Style glabrous, stigma puberulous	Lower half style hairy, stigma puberulous	–
Fruit	Capsule broadly obconic, 7–8 mm long	Capsule narrowly obconic, 6–7 mm long	Capsule sub-globose, laterally compressed, ca. 4 mm long
Flowering time	May to July	May to September	July to September

## Taxonomic description

### Lobelia hongiana

Taxon classificationPlantaeAsteralesCampanulaceae

Q.F.Wang & G.W.Hu sp. nov.

urn:lsid:ipni.org:names:60475915-2

Figure 1 , Table 2

#### Diagnosis.

The new species is distinguished from *L. chinensis* and *L. loochooensis* by its elliptic-obovate or oblanceolate leaves, usually sinuate-dentate margin; hypanthium 2.5–3 mm long, greenish-carmine; calyx lobes 5 or 6, shorter than hypanthium; corolla purplish-white, yellowish- green blotches at the base of lower lobes; glabrous filaments; broadly obconic capsule, 7–8 mm long; flowering time from May to July.

#### Type.

CHINA. Guangxi: Guilin City, Lingui District, Huixian Town, Huixian Wetland, 25°06.158'N, 110°12.563'E, elev. 140 m, 21 June 2016, *G. W. Hu & Z. Z. Li HGW-01120* (holotype HIB!; isotype GXMG!, HIB!).

#### Description.

Herbs, perennial. Stems decumbent, creeping and branched, slender, green or purple, up to 25 cm or higher, glabrous, lower nodes rooted. Leaves alternate, in 2 rows, sessile or petiole up to 2 mm; blade elliptic-obovate, or oblanceolate, thick, 2.2–9 × 1.6–5 mm, glabrous, green or purple beneath, base rounded, obtuse or broadly cuneate, margin usually coarsely sinuate-dentate or occasionally entire, apex obtuse. Flowers solitary, axillary; pedicels slender, 1.3–4.4 cm; hypanthium narrowly obconical, base attenuate, not well distinguished from pedicel, 2.5–3 mm, glabrous, greenish-carmine; calyx lobes 5 or 6, lanceolate, occasionally unevenly bifid, 1.4–2 mm long, shorter than tube, margin with 1 or 2 pair of denticles, occasionally entire. Corolla white, purplish, 9–13 mm, outside glabrous, densely white villous below throat; sub-bilabiate, lobes 5, monomorphic, ovate-lanceolate, 5–7 mm long, longer than the tube, gradually recurved outwards when open, tube split not to base on dorsal side; lower lobes 3, covered sparsely white villous, with yellowish-green blotches at the base; upper lobes 2, glabrous except occasionally covered rarely white villous; filament ca. 4 mm long, adnate to corolla tube on lower third, glabrous, connate above middle, filament tube ca. 1.5 mm; anther tube 1.2–1.5 mm, anther tube bearded with tufts of filiform hairs, ventral anthers bearing two awns, ca. 0.5 mm long. Style enclosed at connate filaments, glabrous, protruded and curved once mature; stigma bifid, puberulous. Ovary 2-locular, ovules numerous. Capsule obconic, apically 2-valved, 6–8 mm long, dehiscing loculicidally, calyx lobes persistent. Seeds narrowly elliptic, terete.

**Figure 1.**
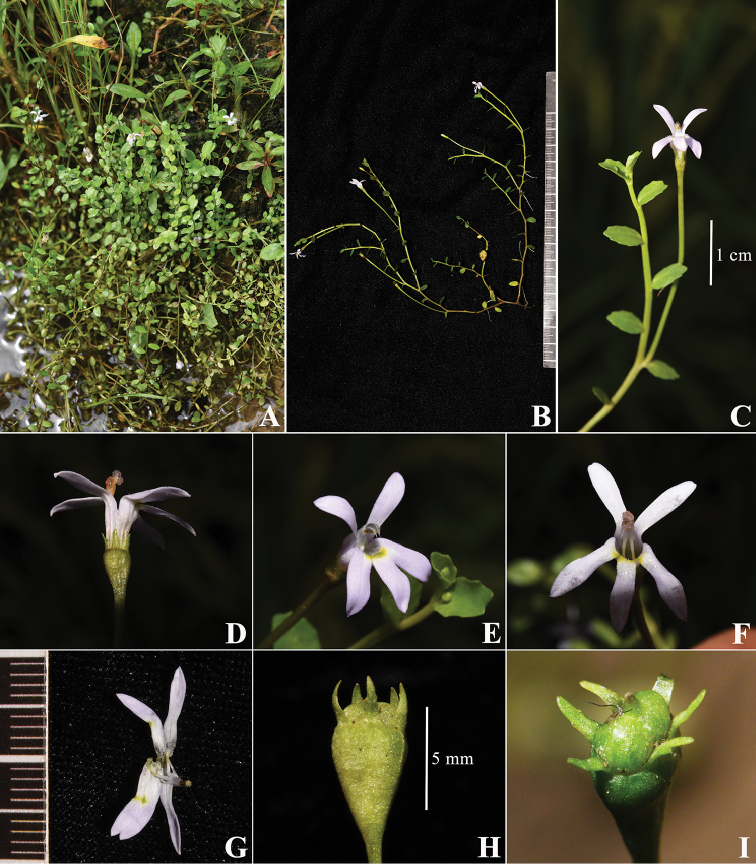
Photos of *Lobelia hongiana* Q.F.Wang & G.W.Hu: morphology. **A** Habitat **B** Part of one individual **C** A stem bearing leaves and a flower **D–G** Flower viewed from different orientations **H–I** Fruit viewed from different orientations.

**Figure 2.**
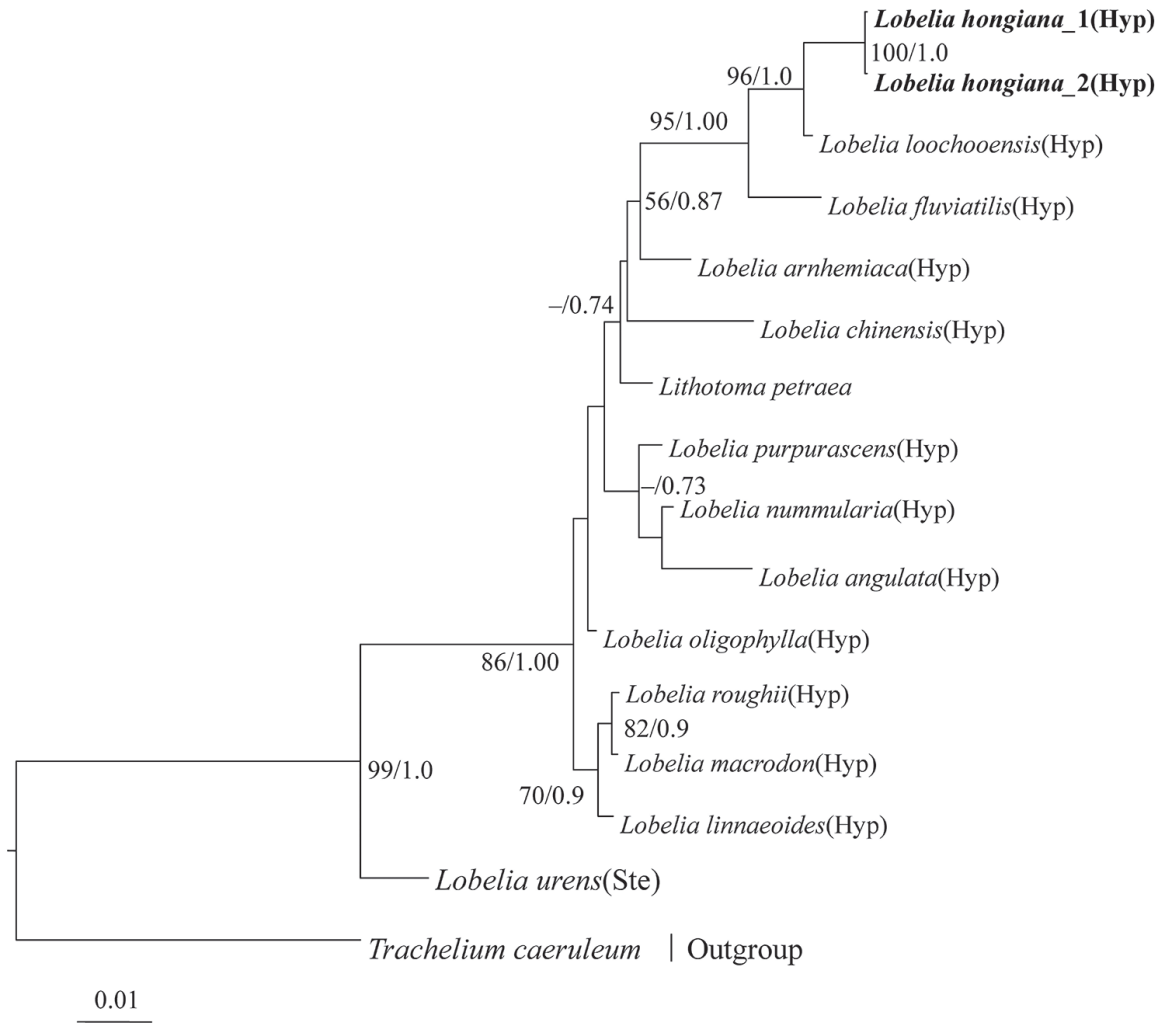
Bayesian tree inferred using the combined ITS, *atpB* and *rbcL* dataset showing the phylogenetic position of *Lobelia hongiana* and its sister species. Maximum likelihood bootstrap values ≥ 50 and PPs ≥ 0.70 are shown at the nodes. The abbreviations, Hyp and Ste, represented the sections of *Hypsela* and *Stenotium*, respectively. The new species is shown in bold.

#### Distribution and ecology.

The new species has been found in Huixian Wetland, Guangxi Zhuang Autonomous Region in China, with only two populations. There is a high probability that *L. hongiana* is also distributed at adjacent areas, given its vegetative propagation traits. Its living environment is wetland and farmland.

#### Phenology.

The new species was found in flower from May to July.

#### Etymology.

Species epithet, “*hongiana*”, is in honour of Prof. De-Yuan Hong who made a significant contribution to the authors’ knowledge of Campanulaceae.

#### Conservation status.

This new species was only found at Huixian Wetland in Guangxi Zhuang Autonomous Region, China, although it might also be distributed in adjacent areas. Until now, about 200 individuals were found in each population. Since there is not enough information on population size and dynamics, an assessment of the current conservation status of this species cannot be given. Therefore, it is suggested that the species be evaluated as Data Deficient (DD) according to the IUCN Red List Categories and Criteria (IUCN 2001).

#### Other specimens examined (paratypes).

CHINA, Guangxi, Guilin City, Lingui District, Huixian Town, elev. 140 m, 8 June 2016, *G. W. Hu & Z. Z. Li HGW-01117* (HIB!)

##### Key to the Lobelia sect. Hypsela in East Asia

**Table:** 

1	Fruit berry, purple-red, ellipsoid or globose; stem villous, rarely glabrous; petiole puberulent	***L. nummularia***
–	Fruit capsule, green, subglobose or obconic; stem glabrous; petiole glabrous	**2**
2	Stem prostrate; leaves thick, pedicel under 2.5 cm long; hypanthium no more than 1 mm long, calyx lobes longer than hypanthium, corolla under 9 mm long; all 5 filaments sparsely hairy; capsule subglobose, no more than 5 mm long	***L. loochooensis***
–	Stem decumbent; leaves thin or slightly thick, pedicel up to 4 cm or longer; hypanthium more than 2.5 mm, calyx lobes not longer than hypanthium, corolla over 9 mm long; not all 5 filaments hairy; capsule obconic, more than 6 mm long	**3**
3	Leaves slightly thick, under 1 cm long, apex obtuse; hypanthium 2.5–3 mm long, calyx lobes 5 or 6, 1.4–2 mm long, shorter than hypanthium, corolla sub-bilabiate, corolla lobes gradually recurved when open, covered sparsely white villous, without vein; filament ca. 4 mm long, glabrous, anther tube 1.1–1.5 mm; style glabrous; capsule broadly obconic, 7–8 mm long	***L. hongiana***
–	Leaves thin, 0.7–2.6 cm long, apex acute or acuminate; hypanthium 3–5 mm long, calyx lobes 5, 3–5 mm long, as long as hypanthium, corolla unilabiate, corolla lobes all spreading in a plane on anterior side, glabrous, with purple veins; filament 6–8 mm long, the two anterior filaments hairy, anther tube 2–2.5 mm; style hairy; capsule narrowly obconic, 6–7 mm long	***L. chinensis***

## Supplementary Material

XML Treatment for Lobelia hongiana
